# Supplementary material for: Melanoma cells adopt features of both mesenchymal and amoeboid migration within confining channels
Source: Sci Rep. 2021 Sep 7;11:17804. doi: 10.1038/s41598-021-97348-7 (PMC8423822; doi:10.1038/s41598-021-97348-7)
Supplement: Supplementary file 1 — Supplementary Legends. [file 41598_2021_97348_MOESM1_ESM.pdf]

Melanoma cells adopt features of both mesenchymal and amoeboid migration within confining channels

Sairisheel R. Gabbireddy[1], Karl W. Vosatka[3], Aram J. Chung[2, 4, 5], and Jeremy S. Logue[3, *]

[1]Undergraduate Research Program, Rensselaer Polytechnic Institute (RPI), 110 8th St, Troy, NY 12180, USA

[2]Department of Mechanical, Aerospace, and Nuclear Engineering, Rensselaer Polytechnic Institute (RPI), 110 8th Street, Troy, NY 12180, USA

[3]Department of Regenerative and Cancer Cell Biology, Albany Medical College, 47 New Scotland Ave, Albany, NY 12208, USA

[4]Present address: School of Biomedical Engineering, Korea University, 02841, Seoul, Republic of Korea

[5]Present address: Interdisciplinary Program in Precision Public Health, Korea University, 02841 Seoul, Republic of Korea

*Corresponding author

loguej@mail.amc.edu

Short title: melanoma cell migration in confining channels

Supplemental Movies

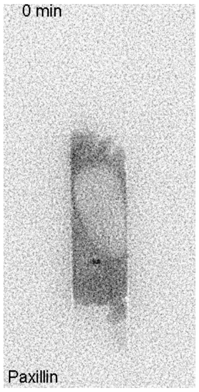

Supplemental movie 1. Time-lapse imaging of a melanoma A375-M2 cell (*phenotype 1*) within a fibronectin coated (10 µg/mL) microchannel with the focal adhesion marker, EGFP-paxillin.

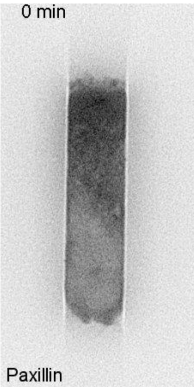

Supplemental movie 2. Time-lapse imaging of a melanoma A375-M2 cell (*phenotype 2*) within a fibronectin coated (10 µg/mL) microchannel with the focal adhesion marker, EGFP-paxillin.

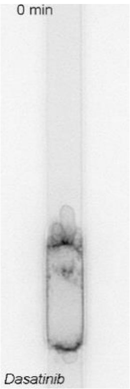

Supplemental movie 3. Time-lapse imaging of a melanoma A375-M2 cell within a fibronectin coated (10 µg/mL) microchannel treated with the Src family kinase inhibitor, Dasatinib (10 µM). Cells were stained with a far red membrane dye.

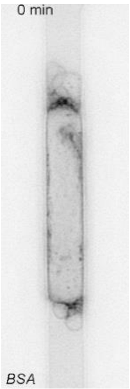

Supplemental movie 4. Time-lapse imaging of a melanoma A375-M2 cell within a BSA coated (1%) microchannel. Cells were stained with a far red membrane dye.

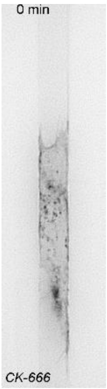

Supplemental movie 5. Time-lapse imaging of a melanoma A375-M2 cell within a fibronectin coated (10 µg/mL) microchannel treated with the Arp2/3 inhibitor, CK-666 (100 µM). Cells were stained with a far red membrane dye.
